# The Importance of Monitoring Sleep within Adolescent Athletes: Athletic, Academic, and Health Considerations

**DOI:** 10.3389/fphys.2016.00101

**Published:** 2016-03-18

**Authors:** Lee Taylor, Bryna C. R. Chrismas, Ben Dascombe, Karim Chamari, Peter M. Fowler

**Affiliations:** ^1^Athlete Health and Performance Research Centre, ASPETAR, Qatar Orthopedic and Sports Medicine HospitalDoha, Qatar; ^2^Sport Science Program, College of Arts and Sciences, Qatar UniversityDoha, Qatar; ^3^Department of Rehabilitation, Nutrition and Sport, School of Allied Health, LaTrobe UniversityMelbourne, VIC, Australia

**Keywords:** sleep disturbance, youth athlete, performance, adaptation, development

Sport academies are the vehicle by which clubs and governing bodies often develop and nurture talented sports people, with their demographic predominately adolescent. Conceptualized initially within Western mass participation sports, academies are now seen across the globe, with a diversity of nationalities, ethnicities, religions, and cultures, encompassing a broad variety of sports.

Substantial prospective capital investment is typically required by academies, balanced by potential future “on-field” (e.g., Olympic Gold Medal, League Champions, etc) and/or “financial” reward (e.g., transfer fee received for a soccer academy graduate). To contextualize, a successful English Premier League soccer academy runs at a cost of ~£2.3 (~$3.3) million per year and has produced graduates worth < £100 (>$143) million since inception, with several players representing their senior national team. The small probability of success within this paradigm, which is mirrored to varying degrees within other sports, has led to a change of ethos within academies. Early academies suffered social and political pressure due to low salaries and “token” educational offerings. Emphasis is now placed on genuine academic and athletic development, in an attempt to ensure that if a successful athletic career is not realized, then employment prospects away from an athletic career are viable. For example, the alignment between the American scholarship system and National Collegiate Athletic Association (NCAA) is well established, with talented athletes attaining full academic scholarships and pursuing Degree level qualifications, with the added prospect of signing professional sport contracts post-graduation, or occasionally during their studies.

Consequently there is increasing competiveness, professionalism, training, and fixture congestion within youth sport, with many suggesting that adolescent athlete rest and recovery is compromised as a result (Bergeron et al., 2015). Worryingly, some youth athletes are now being treated as “commodities” and hence the International Olympic Committee (IOC), amongst others, has called for more diligence to safeguard their psycho-physiological development (Bergeron et al., 2015; Lloyd et al., 2015a,b; Mountjoy and Bergeron, 2015; Mountjoy et al., 2015). The maintenance of appropriate rest, particularly sleep for adolescent athletes was discussed within the recent IOC consensus statement, which stated the need to “design youth athlete development programmes …to mitigate the risk of overuse injuries and other health problems …providing sufficient and regular rest and recovery, to encourage positive adaptations and progressive athletic development” (Bergeron et al., 2015).

The importance of sleep for optimal athletic performance and recovery, cognitive/academic performance, and well-being, together with, reducing injury and illness risk within athletes, including adolescents, has been consistently purported recently (Luke et al., 2011; Milewski et al., 2014; Owens J. et al., 2014; Baert et al., 2015; Bergeron et al., 2015; Diaz-Morales and Escribano, 2015; Fullagar H. H. et al., 2015; Fullagar H. H. K. et al., 2015; Nedelec et al., 2015a; Prather et al., 2015; Thun et al., 2015). These academic, health and performance agendas are central to the modern academy ethos. However, evidence based practices that practitioners and athletes could utilize to maintain sufficient sleep are limited (Halson, 2014, 2015; Fullagar H. H. et al., 2015; Fullagar H. H. K. et al., 2015; Nedelec et al., 2015a,b), especially within adolescent athletes.

Academy athletes regularly undergo extensive physiological monitoring and sport-specific performance testing (Armstrong and McManus, 2011; Barker and Armstrong, 2011). However, given the suggested positive effects of sufficient sleep and its maintenance on several aspects of athletic performance and recovery (Fullagar H. H. et al., 2015; Fullagar H. H. K. et al., 2015), sleep monitoring is not presently given equal importance and attention compared to “typical” physiological capacity testing (e.g., $\overset{{}^{\circ}}{\text{V}}{\text{O}}_{2\text{max}}$). Although attempts were made as early 1966 to examine sleep in athletes (Baekeland and Lasky, 1966), sleep monitoring *per se* within athletes is in its infancy, as is its translation to practice, particularly within adolescent athletes. This is concerning given the susceptibility (Bartel et al., 2015) and incidence of adolescents adopting poor sleep practices and/or suffering disturbed sleep, intentionally or otherwise (Gradisar et al., 2011).

Whilst multi-faceted, and in this opinion article contextualized within a youth athlete paradigm, it is not surprising adolescents suffer sleep disturbances. With advancing adolescence, both positive and negative (e.g., increased nightly screen time, social jet lag, etc.) behaviors develop, which can alter the internal “body clock.” One such alteration is to endogenous melatonin nadirs and zeniths, which can perpetuate delayed sleep phase disorder (DSPD; Eckerberg et al., 2012). Compounding this there is often a lack of synchronization between adolescent sleep-wake cycles (retiring later and rising later, i.e., DSPD) and other age categories, with academic (Escribano et al., 2012; Tonetti et al., 2015) and athletic performance (Carskadon, 2005; Facer-Childs and Brandstaetter, 2015; Thun et al., 2015) known to be chronotype sensitive. Talented adolescent academy athletes are predominately coached within a formalized and timetabled environment. This often involves evening training and matches (with long commutes common), followed by early educational classes with rigid start times (Wolfson et al., 2007; Short et al., 2013; Franckle et al., 2015) the next day. As a result, sub-optimal variation of their biologically preferred sleep-wake cycles can occur (Crowley et al., 2007; Owens J. A. et al., 2014; Hirshkowitz et al., 2015). Adolescents, relative to children and adults, have greater variability in sleep between week days (school, or traditional work days) compared to the weekend, with significantly less sleep on the former (Merdad et al., 2014) facilitating accumulative week day sleep debt (Van Dongen et al., 2003a,b). This training and education induced sleep deficit, coupled with social jet lag (see Touitou, 2013; Rutters et al., 2014; Diaz-Morales and Escribano, 2015; for explanation of concept), is concerning from an athletic/academic performance development perspective. Empirical evidence demonstrates that reduced sleep negatively influences athletic/academic performance and various indices of morbidity. Specifically, reduced and/or disturbed sleep has been shown to negatively influence aerobic (Oliver et al., 2009) and anaerobic performance metrics (Bulbulian et al., 1996; Souissi et al., 2003, 2008, 2013; Skein et al., 2013), increase injury and illness risk (Luke et al., 2011; Milewski et al., 2014) whilst affecting team sport match outcome (Smith et al., 2013). Academically, sleep extension by a single standard deviation led to an increase of 4.85% point in coursework marks in adolescents (Baert et al., 2015) whilst adolescents with insufficient sleep (i.e., DSPD, etc.) have reduced academic achievement and development compared to those with appropriate sleep (Escribano et al., 2012; Diaz-Morales and Escribano, 2015; Sivertsen et al., 2015). Those adolescents with insufficient sleep also increase their risk of various negative health indices and behaviors including, but not limited to; depression, suicidal ideation, anxiety, hyperarousal, increased obesity risk, decreased mental resilience, poor dietary intake, and higher incidences of attention deficit hyperactivity disorder (Kaneita et al., 2007; Nyer et al., 2013; Owens J. et al., 2014; Alibhai et al., 2015; Franckle et al., 2015; Sivertsen et al., 2015). Interestingly within a sample of 287 monozygotic twins, those who slept less exhibited lower levels of self-control and more depressive mental health symptoms (Barnes and Meldrum, 2015) despite identical genetics and shared environmental influences. Thus chronotype and/or sleep disturbances, even between genetically identical individuals with shared environmental influences, can alter a range of academic (Escribano et al., 2012; Diaz-Morales and Escribano, 2015; Tonetti et al., 2015), physical performance (Fullagar H. H. et al., 2015), and mental health indices (Kaneita et al., 2007; Nyer et al., 2013; Owens J. et al., 2014; Sivertsen et al., 2015). Such changes can occur either acutely and/or chronically in adolescents, which warrants the monitoring of sleep behaviors in such a population (Shochat et al., 2014).

Psychological overload and resultant heightened anxiety has been reported within youth athletes, which may stem from, at least in part, non-achievable expectations and demands (Malina, 2010; DiFiori et al., 2014) which may in some instances be parent derived (Harwood and Knight, 2009). Anxiety is acknowledged as a central, albeit multi-faceted factor relative to disturbed sleep within adolescents, the general population, and athletes (Staner, 2003; Papadimitriou and Linkowski, 2005; Mellman, 2006; Juliff et al., 2015; Lastella et al., 2015a,b; Romyn et al., 2016). However, whether anxiety induces sleep disturbance or sleep disturbances result in heightened existing anxiety and/or reduced resilience to anxiety, demonstrates individualization within and between the aforementioned populations. Parallel to this, sleep medication use (with and without prescription) within adolescents is of medical concern, with notable ethnic, gender, mental health, academic, and socio-economic predisposing factors present (McCabe et al., 2011; McCabe and West, 2014; Rigg and Ford, 2014; Boyd et al., 2015; Diaz-Morales and Escribano, 2015; Liakoni et al., 2015; Grandner et al., 2016). Data indicates a ~25% incidence of medication misuse within adolescents from at least one of the following medication classes; pain, stimulant, sleeping, and anti-anxiety (McCabe et al., 2011). Specific to adolescent athletes, a report from the NCAA revealed that 10.3% of miscellaneous substance abuse was accounted for by sleep medication, with sport-specific differences present (Taylor et al., 2016). For example, 18.2% of male swimmers (the highest within the report) used such medication (Rexroat, 2014). Therefore, aside from an academic and athletic development perspective, monitoring of academy athletes sleep, and associated behaviors, requires consideration from guardian, pastoral, and ethical perspectives (e.g., medication use, mental health, other non-desirable behaviors, and traits, etc.).

Given the global distribution of academies, and the diversity of nationalities and religions within them, cultural differences are evidently present. Islamic fasting for example is distinct from regular voluntary or experimental fasting (Bahammam et al., 2014) and reduced sleep duration during Ramadan fasting compared to non-fasting has been reported (Bahammam et al., 2013). Indeed, compared to Western Societies, Arab Societies tend to “have a culture associated with a lifestyle that does not promote sufficient hours of sleep each night” (Merdad et al., 2014). For example, data from Saudi Arabia indicates that ~30% of adolescents sleep < 5 h per night, with an average of 6.8 h in “night sleepers” and 8.5 h in “day sleepers.” Of particular concern is that ~10% of this sample demonstrated reverse sleep-wake cycles outside of Ramadan (i.e., they slept during the day; Merdad et al., 2014). Relative to The National Sleep Foundations recommendations (Hirshkowitz et al., 2015) of 9–11 h (6–13 years) and 8–10 h (14–17 years) sleep per night for adolescents, those of comparable ages from Greece (6.9 h; Lazaratou et al., 2005), Iran (7.7 h; Ghanizadeh et al., 2008), Hong Kong (7.3 h; Chung and Cheung, 2008), Israel (7.3 h; Shochat et al., 2010), and South Africa (7.3 h; Reid et al., 2002) exhibit mean sleep durations below these recommendations (Merdad et al., 2014). A plausible explanation for this could be the increase in screen time (televisions, laptops, tablets, smart phones, eReaders, etc.) and social media use (Cain and Gradisar, 2010; Shochat et al., 2010) across society (Gamble et al., 2014; Halson, 2015), particularly within adolescents (Peiro-Velert et al., 2014; Pieters et al., 2014; Hale and Guan, 2015). Social media use and associated screen time can contribute to a “fear of missing out,” which can perpetuate an “on call” psychological state, evidently counterintuitive to appropriate sleep (Halson, 2015; Lister-Landman et al., 2015). Such use of technology negatively influences desired sleep parameters, subsequent alertness and attention whilst also increasing next day caffeinated beverage consumption in an attempt to acquiesce the previous night's sleep disturbance induced drowsiness, this likely negatively influences subsequent evening sleep dependent upon time of consumption (Cain and Gradisar, 2010; Gamble et al., 2014; Dimitriou et al., 2015). These issues, discussed throughout this opinion piece, regarding sleep disturbances within adolescents, discreetly, or in various combinations, function to hinder achieving the quantity and quality of sleep endorsed by The National Sleep Foundation for adolescents (Hirshkowitz et al., 2015).

Although sleep disturbances and undesired sleep-wake patterns are evident in young people across the globe, the inter- and intra-continent variation across, and between North America (Liu et al., 2005), Asia (Chung and Cheung, 2008; Merdad et al., 2014), Europe (Loessl et al., 2008), and Australasia (Short et al., 2013) ensures a “one size fits all” solution to rectify insufficient sleep is not externally valid, with individualization, perhaps even within the same academy likely required (Fullagar and Bartlett, 2016). Indeed, this individualized solution cannot be implemented without quantification of the problem. The problem is difficult to detect through parents/guardians, since between 40% (Meltzer et al., 2013) and 60% (Amschler and McKenzie, 2005) of parents are unaware of their adolescents undesirable sleep patterns. Therefore, sleep monitoring in adolescent athletes should be externally valid (i.e., non-invasive and easy to implement away from a laboratory, training ground, etc.), portable (i.e., for away fixtures and training camps) whilst possessing high validity and reliability compared to polysomnography (PSG). Validation studies comparing wrist activity monitors with PSG (n.b. the gold standard for monitoring sleep) report high correlations for sleep duration (i.e., 0.84–0.90) and moderate-to-high correlations for wake time within sleep (i.e., 0.53–0.76; Kosmadopoulos et al., 2014; Sargent et al., 2015). However, existing actigraphy data in this regard is skewed toward older- and post-adolescent athletes with recent data (~17–25 years) seen across 5 days to 6 weeks training or competition phases (Sargent et al., 2014, 2015; Killer et al., 2015; Kölling et al., 2015b; Lastella et al., 2015a,b; Shearer et al., 2015; Dennis et al., 2016; Romyn et al., 2016), with only a single data set (~24 years) collected across a full season (Dennis et al., 2016). There is a paucity of season-long sleep monitoring data from school age (8–18 years) adolescent academy athletes in addition to cultural comparisons (i.e., Arab compared to Western academies). Wrist actigraphy is therefore recommended as a practically administrable tool to quantitatively monitor sleep whether the athlete is at their home, or not. Furthermore, an appropriately composed questionnaire could enrich the wrist actigraphy derived data however, there is not currently a ubiquitously adopted “sleep quality” questionnaire administered by practitioners. Practitioners, researchers and athletes should work together to develop and validate such an appropriate sleep questionnaire (validated against actigraphy) for adoption across practice, advancing recent efforts related to team sports (Kölling et al., 2015a; Fullagar et al., 2016).

A reduction in adolescent sleep has occurred over the last century (Matricciani et al., 2011). Specifically, within the past two decades, a mismatch has developed between the perception of adequate sleep and adolescents globally reported actual sleep durations (Keyes et al., 2015). Moreover, this reduction has been overtly modified by socio-economic, racial, ethnicity and religious factors (Keyes et al., 2015). The contextualized empirically informed paradigm presented within this opinion article strongly suggests there is an obligation of academies, practitioners, athletes, and their parents to ensure academic and athletic development are supported by appropriate sleep. This can only be realized if appropriate sleep monitoring (utilizing the practical methodological recommendations stated earlier) is embedded within academy programmes, and is embraced by the athlete and their support network. Implementation, adoption and athlete adherence to such monitoring is dependent upon appropriate interaction between medically qualified staff, athletes and practitioners, and tri-partite evidence informed education within this axis (Taylor et al., 2016). Adolescent academy athletes must be made overtly aware of the consequences of insufficient sleep on their holistic development, with education the most viable vehicle to facilitate this awareness and adherence (Burgess and Naughton, 2010). These academy sleep issues may be particularly important in the Middle East as cultural factors may result in very poor sleep (as detailed above) compared to age matched controls from other regions (Merdad et al., 2014). Without such a change in practice, not only are long term athletic and academic successes jeopardized, but serious pathophysiologies, health issues, risk taking behavior, and poor quality of life are all dangerously increased for the adolescent athlete (Owens J. et al., 2014).

## Author contributions

LT, BC, BD, KC, PF—contributed to conceptualization, drafting, and critical appraisal of the opinion article.

### Conflict of interest statement

The authors declare that the research was conducted in the absence of any commercial or financial relationships that could be construed as a potential conflict of interest.
